# Development of an educational intervention for patients with Irritable Bowel Syndrome (IBS) – a pilot study

**DOI:** 10.1186/1471-230X-9-10

**Published:** 2009-02-04

**Authors:** Gisela Ringström, Stine Störsrud, Sara Lundqvist, Berndt Westman, Magnus Simrén

**Affiliations:** 1 Dept of Internal Medicine, Institute of medicine, Sahlgrenska Academy, University of Gothenburg, Gothenburg, Sweden

## Abstract

**Background:**

Many IBS patients experience that they receive limited information and that the health care system does not take their complaints seriously. We aimed to develop a structured patient education, an 'IBS school', and investigate if the efficacy could be evaluated in terms of improved knowledge, symptom severity and health related quality of life (HRQOL).

**Methods:**

The IBS school consisted of six weekly two hour sessions in a group setting. Five different health care professionals were responsible for one session each. Questionnaires covering patients' experience of the education, perceived knowledge about IBS, gastrointestinal symptoms, and HRQOL, were used for evaluation at baseline and at three, six, and twelve months after education.

**Results:**

Twelve IBS patients were included. The patients were overall satisfied with the IBS school. In line with this, the gastrointestinal symptoms, HRQOL, and perceived knowledge about IBS improved significantly after the education.

**Conclusion:**

An IBS school seems to be a proper method to meet the patients' need of information about IBS and also to improve the patients' gastrointestinal symptoms, HRQOL, and knowledge about IBS. Further controlled studies are now needed in larger numbers of patients to confirm these preliminary results in order to implement this intervention in clinical practice.

## Background

Irritable Bowel Syndrome (IBS) is one of several functional gastrointestinal (GI) disorders, and has a prevalence of 10–20 % in western countries [1,2]. The disorder is characterized by abdominal pain and/or discomfort, associated with disturbed bowel habits, as defined by diagnostic criteria, such as the Rome II [3] and Rome III criteria [4]. So far, the pathophysiological mechanisms are not clearly known and medical treatment options are limited. Moreover, overlap with other functional gastrointestinal disorders is common, resulting in a great symptom burden for some patients [1], even though IBS, from a medical point of view, is a harmless disease [5]. It has previously been reported that psychological symptoms are more common in IBS patients compared with the normal population, but not considered to be the cause of the disease [6]. Moreover, health related quality of life (HRQOL) is reported to be poor among IBS patients compared with the general population [7], and equally bad, or even worse, compared with other medically more severe diseases [8].

Many IBS patients experience disabling symptoms and negative interference in daily life, and feel that they are not taken seriously in their contact with the health care system [9-11]. This could partly be due to limited knowledge about functional GI disorders among health care workers [12]. IBS patients often ask for explanations and education about their disease rather than a pill to cure their symptoms [13], and educational guidelines can improve the management of IBS patients in primary care [14].

Educational interventions have been performed and found to be useful for patients with different kinds of chronic diseases such as inflammatory bowel disease [15], diabetes [16], generalized chronic musculoskeletal pain [17], rheumatic diseases [18], asthma [19], and coronary artery disease [20]. Some educational interventions have also been evaluated in IBS patients with satisfying results [21-23]. Also in connection to a first visit to a gastroenterologist, an extra 15 minutes of reassurance can reduce self-perception of impairment in daily functions in IBS patients [24].

An educational intervention performed in a group setting offers the patients possibilities to meet other people with similar symptoms and difficulties, in order to achieve confirmation that they are not alone. It also provides an opportunity to share experiences with each other within the group [25]. It has been demonstrated that a mixture of information giving, teaching and counselling should be used when educating patients [26]. Moreover, the education has to be flexible enough to fit all patients, but also structured enough to enable evaluation. When evaluating interventions such as patient education it is important not only to measure knowledge gain, but the evaluation should also include indicators of adherence to the health-care regimen and health outcomes [27].

The primary aim of this pilot study was to develop an education for IBS patients, an 'IBS-school', and evaluate if this is a proper way to provide information to patients. Moreover, we wanted to investigate if the effects of this intervention could be evaluated in terms of perceived knowledge about IBS, severity of GI symptoms and HRQOL.

## Methods

### Patients

Sixteen consecutive patients with IBS according to the Rome II criteria [3] attending the GI out patient clinic at Sahlgrenska University hospital in Gothenburg, Sweden, were asked to participate in this pilot study. Patients with an organic GI disease and/or with another disease potentially affecting the GI symptoms were excluded. Likewise, patients with severe psychiatric disease were excluded due to potential problems with taking part in a group intervention. The patients received written information by mail together with an invitation to an appointment with a nurse specialized in functional GI disorders and also responsible for the study (GR). The nurse conducted an individual 30 minutes interview with each patient before inclusion. The aim of this interview was to collect information regarding medical history, symptom pattern, impact on daily life, most bothersome symptom, demographic data, and information regarding what the patients expected to gain from the patient education. The interviews also served to give the patients additional information about the study. All subjects gave written informed consent and the ethics committee of Göteborg University approved the study.

### IBS-school

The contents of the patient education were selected based on results from a previous study at our unit, investigating the need of information and education in IBS patients (G Ringström et al. accepted for publication in Gastroenterology Nursing 2008). This study, in which 86 IBS patients completed a questionnaire regarding knowledge of IBS, demonstrated that the patients mainly wanted information about what they can do in order to reduce their symptoms, treatment options, and causes of symptoms. Many patients had a lack of knowledge in areas related to pain/discomfort, the role of diet, the risk that IBS will turn into a serious disease, and the diagnostic work-up in IBS. The design of the IBS-school was based on the Self-Efficacy Theory, which contains four specific efficacy-enhancing mechanisms: skills mastery, modelling, reinterpretation of physiological signs and symptoms, and persuasion [28,29]. All these mechanisms were considered to be important parts of the IBS-school. Especially, patients were encouraged to try new treatments and lifestyle changes step by step, and evaluate the effects on symptom severity appropriately, before trying something new. Modelling was used by encouraging the patients to share their own experiences of methods and strategies found to be useful in their attempts to manage symptoms. The General Theory of Nursing [30] also formed a frame for the IBS-school. According to this theory, self-care is what individuals do themselves to regulate their own functioning and well-being. We assumed that an increased level of disease related knowledge would increase the ability to perform self-care activities and lead to improvement of symptoms and well-being. Moreover, the IBS-school was based on a biopsychosocial model considered to be important in functional GI disorders [31]. The education consisted of six weekly meetings in a group setting with five to seven patients in each group. Five different health care professionals were involved in the education in order to cover a wide spectrum of IBS related issues. Each of the professionals held one two hour-session where discussion within the group was encouraged. The patients also received general written information about IBS containing relaxation methods and dietary advice, which is routinely used at our hospital. The patients were provided with handouts, containing the pp slides used during the sessions, except for the last two sessions, where no slideshows were used. The written information and handouts were also given to patients who missed a session. The nurse was responsible for both the first and the last meeting, and also participated in all sessions in order to answer questions that were outside the topic for the present session.

#### Session one

was organized by the nurse (GR) and served to create a comfortable atmosphere as well as an introduction to the entire education. Issues such as what it is like to live with a chronic disease and cope with bothersome symptoms in daily life were discussed, including questions about acceptance of and adaptation to the disease. A brief overview of GI anatomy and physiology was given, as well as elementary facts about IBS. Since medical treatment options are limited we stressed that it is of great importance for patients to get reassurance and realize that they have the capacity to influence their symptoms. The patients were also informed that lifestyle changes, big or small, can be required in order to improve symptoms. It was clarified to the patients that there are no standard methods to be used for IBS symptoms in general, but that they have to identify what they can do in their specific situation, and that health care providers can support them in doing so [32].

#### Session two

was led by a gastroenterologist (MS). Information was given about pathophysiological mechanisms in IBS and the scientific progress that has occurred over the last years. We further emphasized that IBS, from a medical point of view, is not a dangerous disorder, but that it has profound negative impact on quality of life. Since the cause of IBS is not clearly known, a symptom based explanatory model was used, with the aim to provide understandable explanations to the different symptoms. Some factors that can improve and/or worsen symptoms are known, and these were presented to the group. During this session another topic was the overlap with other functional GI disorders and extra intestinal symptoms. Also difficulties in the meeting between patients and health care providers were debated, and reasons for this were explained and discussed. Moreover, information was given about medical and other treatment options, both the ones we have today and potential future options. Indications for investigations to exclude other diagnoses than IBS were also clarified [5,33].

#### Session three

The dietician (SS) discussed food related issues in general and for IBS in particular. It has been reported that a large proportion of IBS subjects limit or exclude food items from their diet [34]. General advice was provided and the patients were encouraged not to avoid food. It was stressed that it might be useful to reduce the intake of some food items, but exclusion diets should only be tried in rare cases and under strict supervision. The focus was not on what the patients eat, but rather on how and when they eat. Regular eating habits are important, and three main meals and two to three snacks were recommended. Explanations regarding symptoms induced by food intake were provided, and even though a meal might induce GI symptoms, the participants were informed that the GI tract will not be damaged, in contrast to patients with celiac disease, ingesting food containing gluten. The importance of gas producing food items, including fibre intake, the importance of lactose and fructose intolerance and cooking methods, were also considered to be important issues to discuss in the group [35,36]. The subjects were also informed that probiotics could be tried since they might improve symptoms in subgroups of patients [37].

#### Session four

The physiotherapist (SL) focused on the link between body and mind, including items such as breathing pattern, body awareness, stress, and pain. A simplified lecture was given about the autonomic nervous system in order to illustrate connections between different parts of the body and how knowledge about this can help to improve symptoms. Stress is known to increase symptoms in IBS [38,39], and the importance of identifying and eliminating stressors in daily life was discussed within the group. Evidence that IBS patients can benefit from physical activity [40], was also presented. Moreover, since relaxation can improve symptoms in IBS [41], a short relaxation practice was performed during this session.

#### Session five

was held by the psychologist (BW), and the title of this session was "Despite IBS, is it possible to live a good life?" Many patients are frustrated about the limitations they experience in their daily life due to GI and extra intestinal symptoms. The importance of verbalizing these feelings of frustration in order to manage symptoms in daily life was discussed. Difficulties to talk about GI symptoms with family and friends are common, and this session also focused on accepting having a chronic disorder. This session was held in an open manner to allow spontaneous discussions between the patients in order to share experiences of more successful coping strategies. Psychological treatment is known to be efficient for symptom improvement in IBS [31], and the patients were provided with information about different forms of psychological treatment.

#### Session six

The final meeting was held by the nurse (GR). The aim of this session was to summarize the entire course. The patients were asked to recall and reflect upon what they had learnt and how they thought they could use this new knowledge. Identifying the knowledge one has and the implementation of it into practical use in daily life is an active process, which is supposed to continue for a long time after the education. The patients were also asked if there was anything that needed to be explained more thoroughly. For patients who had missed a session, the last meeting also served as a forum to answer questions. Many patients are interested in participating in self-help groups [25], and information about the Swedish GI patient support group (Riksförbundet för magtarmsjuka, RMT), working in the entire GI field, was also provided.

### Questionnaires

The patients completed self-administered questionnaires before starting the education (baseline) and at three, six and twelve months after the start of the education, i.e. the evaluation at three months was approximately six weeks post intervention. The baseline questionnaires were completed in connection with the interview, and all follow up questionnaires were mailed to the patients together with a return envelope.

#### Course evaluation

The education was evaluated by the patients, and this was done for each session separately, as well as for the entire course. For this we used a seven graded scale (1 = 'bad', 7 = 'good') with one question for each session, worded 'How did you experience session one?' There was also a possibility to make comments in relation to each question. Additionally, there was one question evaluating the entire course. The evaluation sheet was given to the patients during the first session in order to enable the patients to do their evaluation immediately after each session. The evaluation form was collected anonymously at the end of the last session.

#### Perceived knowledge

The patients were asked to rate their perceived knowledge about IBS on a visual analogue scale (VAS). Two separate scales were used, one for perceived knowledge, and one for their satisfaction with that knowledge. The scales comprised a 100 mm straight line with the extremes labelled 'No knowledge at all' and 'Very much knowledge', and 'Not at all satisfied' and 'Very satisfied'. This measurement has been used previously [11].

#### Individual goal

The patients stated their individual goal with the education at baseline. This was an open question and it was evaluated at the three month follow up by asking the patients if they felt that they had reached their goal completely, partially, or not at all.

#### IBS Severity Scoring System (IBS-SSS)

was developed to rate IBS symptoms and extracolonic features scored on a VAS (0–100 mm). The higher the score, the more severe the symptoms. The overall IBS score is calculated from five items, pain severity, pain frequency, abdominal bloating, bowel habit dissatisfaction and life interference, ranging from 0 to 500. An overall extra colonic score is calculated from ten items, namely nausea/vomiting, early satiety, headaches, backache, excess wind, heartburn, bodily aches, urinary symptoms, thigh pain and lethargy, also ranging from 0 to 500 [42,43].

#### Short Form-36 (SF-36)

was used to assess HRQOL. It is a generic HRQOL measure with eight multi-item subscales (35 items in total), including physical functioning, role limitations due to physical problems, bodily pain, general health perceptions, vitality, social functioning, role limitations due to emotional problems, and mental health. A Physical Component score (PCS) and a Mental Component score (MCS) can be calculated and used as summary scores. Raw scores are transformed into a scale from 0 (worst possible health state) to 100 (best possible health state) on each of the eight subscales [44-46].

### Data Analysis and statistics

Comparisons were made between baseline and all follow up evaluations, namely at three, six and twelve months after the start of education. SPSS version 14.0 was used for the statistical analyses. Since the results from the questionnaires should be considered as ordinal data, comparisons were made with a non-parametric method (Wilcoxon Signed Ranks Test). Results from the questionnaires are given as both median values and Interquartile Range (IQR) (Median (IQR)), and as mean values and standard deviation (SD) (mean (SD)). Unless otherwise stated, significance was accepted at the 5% level (p < 0.05)

## Results

Sixteen patients were approached for the study. Three patients, who attended the interview with the nurse, could not, due to practical difficulties at work or social reasons, participate in the education. One patient started the education but was hospitalized due to severe IBS after two sessions and could not complete the educational program and is therefore not included in the analysis. Twelve patients completed the education (mean age, 37 years; range 26–56 years; 9 females and 3 males). The mean duration of the GI symptoms was 15 years (range 2.5–51 years). Nine of the patients were married or cohabiting and three were living alone. Based on the Rome II criteria, two patients were diarrhoea predominant (IBS-D), four were constipation predominant (IBS-C), and six had alternating bowel habits (IBS-A) (table 1).

**Table 1:** Demographic data

Variable	n = 12
Gender: Male/Female (n)	3/9
Age years (mean ± SD)	37 (± 11)
Symptom duration years (mean ± SD)	15 (± 13)
IBS subtype (n (%))	
IBS-D	2 (17)
IBS-C	4 (33)
IBS-A	6 (50)
Marital status: Cohabiting/Single	9/3

IBS subtype D = Diarrhoea, C = Constipation, A = Alternating.

The patients were divided into two groups, seven (four females) in the first, and five (all females) in the second. Four patients participated in all six sessions, three patients in five, four patients in four, and one patient in three sessions. Those who missed one or two sessions stated that the reasons were difficulties taking time off work or finding a baby sitter. The patient who missed three sessions claimed that her IBS symptoms were so severe those days that it was impossible for her to attend the class.

### Patients' evaluation of the education

Each session, as well as the entire education, was evaluated by the patients on a seven graded scale. They were overall satisfied with the content and the way the course was organized and performed (table 2). The majority gave positive comments on the mix of health care professionals involved, since they realized that different factors in their lives contribute to their health outcome. Moreover, some patients expressed that meeting other IBS patients was very useful. For some patients this was the first time talking to anyone, except for health care providers, about their symptoms and difficulties, and they felt that they could view their situation from a different perspective after the education. There were also negative criticism given by some patients, regarding too little time for questions and that some participants took too much time for individual issues.

**Table 2:** Evaluation of the structured patient education on a seven graded scale.

Session	Mean (± SD)	Median	Minimum	Maximum
1. Introduction	6.75 (0.7)	7	5	7
2. Pathophysiology	6.58 (0.8)	7	5	7
3. Dietary advice	6.38 (1.1)	7	4	7
4. Stress and relaxation	5.64 (2.0)	7	1	7
5. Psychological factors	5.64 (1.7)	6	2	7
6. Summary	6.40 (1.1)	7	4	7
Entire course	6.42 (0.7)	6.5	5	7

### Knowledge about IBS

**Figure 1:**
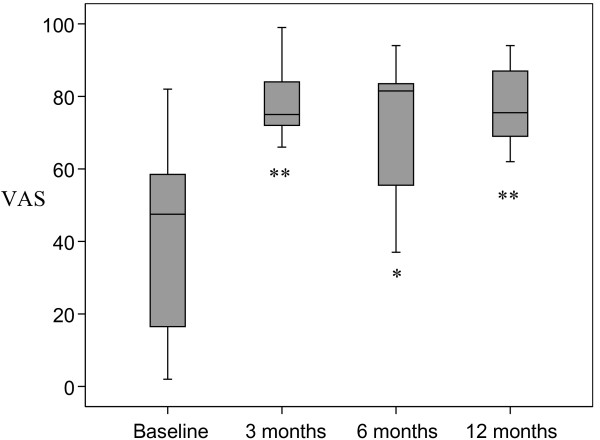
**Perceived knowledge about IBS was measured with a Visual Analogue Scale (VAS) and demonstrated a significant improvement compared to baseline**. * <0.05 **p < 0.01

**Figure 2:**
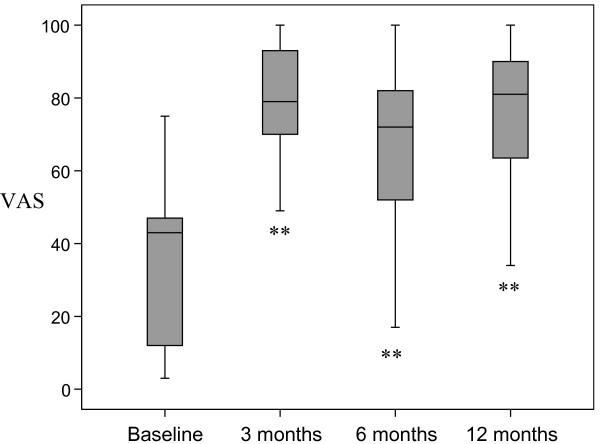
**Satisfaction with perceived knowledge about IBS was measured with a Visual Analogue Scale (VAS), and demonstrated a significant improvement compared to baseline**. **p < 0.01

There was a statistically significant increase in perceived knowledge about IBS after the education compared to baseline, which was maintained throughout the follow up period of twelve months (figure 1). The satisfaction about the knowledge was also increased and maintained in a similar fashion (figure 2).

### Evaluation of the individual goal with the education

The patients formulated individual goals such as, 'An opportunity to meet others in a similar situation', 'To learn about facts regarding diet, physical activity and understand what happens in the gut', 'To obtain knowledge leading to confidence and ability to explain to friends and family about IBS', and 'To get control over symptoms'. Six patients (50%) experienced that they had reached their individual goal completely, five patients reached their goal partially and one patient did not reach the goal at all. Goals that were completely met dealt with obtaining knowledge leading to confidence and ability to explain to friends and family about IBS, and the opportunity to meet other patients in a similar situation. Another goal, "to get control over symptoms", was only partially met. Some patients expressed that it would probably take longer than three months to meet this goal.

### Gastrointestinal symptoms

GI symptom severity was reduced after the education as indicated by lower scores on IBS-SSS. The IBS symptom score was significantly reduced at three (p < 0.05) and six months (p < 0.05) after the IBS school, and was still, but not significantly, improved at the twelve months follow up, compared to baseline. The extra colonic scores also indicated less severe symptoms during the entire follow up period, although this failed to reach statistical significance (table 3). One patient failed to complete the questionnaire at the three-month follow up.

**Table 3:** The gastrointestinal symptom severity, measured with IBS-SSS, at three, six and twelve months after the IBS-school compared to baseline.

	IBS-score		Extracolonic score	
	Median (IQR)	Mean (SD)	Median (IQR)	Mean (SD)
Baseline (n = 12)	284 (233–369)	293 (80)	211 (143–241)	191 (74)
3 months (n = 11)	253 (183–337)*	244 (109)	162 (129–238)	171 (81)
6 months (n = 12)	240 (178–326)*	246 (103)	170 (145–233)	177 (66)
12 months (n = 12)	269 (159–346)	252 (105)	161 (142–223)	168 (78)

IBS-SSS = IBS Severity Scoring System. * = p < 0.05

### Health related quality of life

Statistically significant improvements in HRQOL were demonstrated after the education compared with baseline on several of the domains on SF-36. This improvement was seen for both the physical and mental summary scores (table 4). One patient failed to complete the questionnaire at the three-month follow up.

**Table 4:** Health related quality of life, according to SF-36, at three, six and twelve months after the IBS school compared to baseline.

Variable	Baseline (n = 12)		3 months (n = 11)		6 months (n = 12)		12 months (n = 12)	
	Median (IQR)	*Mean (SD)*	Median (IQR)	*Mean (SD)*	Median (IQR)	*Mean (SD)*	Median (IQR)	*Mean (SD)*
**MCS**	32(21–42)	*31(11)*	44(33–55)*	*41(16)*	40(32–55)*	*41(13)*	36(21–54)	*37(17)*
**PCS**	37(32–42)	*38(8)*	42(33–47)*	*41(9)*	41(32–49)	*40(10)*	37(26–49)	*37(13)*

SF-36 = Short Form-36, MCS = Mental component score, PCS = Physical Component score.

* p < 0.05

## Discussion

In this pilot study we found the concept of an IBS-school to be a useful method to provide information to IBS patients. The patients expressed great satisfaction with the education, and the majority reached their individual goal with the intervention. The perceived knowledge was significantly increased and maintained throughout the twelve months of follow up. Some improvements were also found regarding GI symptom severity and HRQOL, but statistical significance was not seen for all comparisons, probably due to the small sample size in this pilot study.

Our findings regarding increased perceived knowledge about IBS and satisfaction with that knowledge after the IBS-school, are in line with several other studies reporting that patients gain knowledge about their disorders after education [15,19,20]. Our patients also experienced that they reached their individual goal during the education, which correlates well with an enhanced ability to cope with symptoms in daily life. However, written information only, has also been shown to be effective in the treatment of IBS patients in order to increase self-care activity and decrease primary care visits [47].

The IBS-school as a method to provide information was very much appreciated by the patients as indicated by high scores on the evaluation form. A recent study regarding educational needs in IBS patients has shown that patients are mostly interested in learning how to manage their condition on a daily basis [48]. This is in concordance with the aim of our intervention. The patients also experienced a great benefit from meeting other patients, sharing similar symptoms and difficulties, and discussing these issues with them, which is in accordance with a previous study [25]. The importance of interaction between patients is also supported by another recent study, where educated migraine patients successfully passed on information to other migraine patients [49].

A positive effect of the IBS-school on GI symptoms and HRQOL, as shown in this study, is well in line with some previous studies. Heitkemper et al. showed that both an eight-session program and a one-session version, significantly improved HRQOL in women with IBS, compared with controls. GI symptoms were reduced, but this improvement reached statistical significance in the eight-session program only [21], suggesting that patient education should be performed with multiple meetings. Saito et al. showed that a one-session educational class for IBS patients improved health-promoting lifestyle behaviour and to some extent also symptoms [22]. A Swedish study reported some benefits in terms of symptom and HRQOL improvement, as well as a reduction of health care consumption among IBS patients following a four-session education [23]. All available results point in the same direction, telling us that many IBS patients will benefit from an educational intervention. Further development and evaluation of different forms of education for IBS patients seems to be of great importance. Moreover, patient education in the form presented in our study could be considered time consuming and costly, why future studies on cost effectiveness are needed. Finally, it seems to be appropriate to evaluate the effects of patient education in terms of perceived knowledge about IBS, GI symptom severity and HRQOL.

There are some limitations with this study. One factor that might play a role in patient education is whether the patients are recruited from secondary/tertiary care or from primary care. Secondary/tertiary care patients have higher intensity of abdominal pain and higher amount of interference with daily activities [50] compared with primary care patients. Furthermore, primary care patients also have better HRQOL, less psychological symptoms and fatigue compared with secondary/tertiary care patients [51]. These factors might influence the results of our study. Moreover, the results regarding HRQOL and GI symptoms might not be very convincing since some of the improvements failed to reach statistical significance. However, this may partly be explained by the small sample size. Importantly, the changes in GI symptoms and HRQOL were uniform and in the direction of improvement for the majority of the patients. It is now important to perform randomized controlled studies in larger groups of patients in order to confirm these positive results. Moreover, education might be even more effective if the intervention is performed close to the onset of symptoms and diagnosis [52]. The majority of the patients in our study had long standing symptom duration. However, the present study was mainly aimed to evaluate the concept 'IBS-school', and was therefore performed in a small sample of patients from secondary/tertiary care.

## Conclusion

To conclude, it seems that this kind of intervention is a satisfactory method to provide patients with information and knowledge about IBS, as well as an opportunity for IBS patients to share experiences with each other.

Further studies are needed to evaluate effects on GI symptoms and HRQOL in larger number of patients, as well as in primary care patients and in patients with shorter symptom duration. Moreover, future studies should include a valid comparison with different groups receiving some other intervention in order to confirm the efficacy of our structured patient education. Furthermore, extra intestinal symptoms, coping resources, health care consumption and work absenteeism due to illness, are other variables worth measuring when evaluating this kind of intervention. Finally, these future studies are needed before implementing this intervention into clinical practice.

## Abbreviations

IBS: Irritable Bowel Syndrome; GI: Gastrointestinal; VAS: Visual Analogue Scale; HRQOL: Health Related Quality of Life; IBS-SSS: IBS Severity Scoring System; SF-36: Short Form-36; PCS: Physical Component Score; MCS: Mental Component Score.

## Competing interests

The authors declare that they have no competing interests.

## Authors' contributions

GR and MS shared the responsibility for the overall planning of the IBS-school and this pilot study. All authors participated in the design of the study and all authors read and approved the final manuscript. GR planned and performed the first and last session of the IBS-school and was responsible for the collection of questionnaires and all the analyses performed in the study. MS planned and performed the second session, SS planned and performed the third session, SL planned and performed the fourth session, and BW planned and performed the fifth session.

## Pre-publication history

The pre-publication history for this paper can be accessed here:

http://www.biomedcentral.com/1471-230X/9/10/prepub
